# The effectiveness of therapeutic craft-making activities in treating lower-third forearm fracture: study protocol for a randomized controlled trial

**DOI:** 10.1186/s13063-024-08008-w

**Published:** 2024-03-12

**Authors:** Rui Fen Teoh, Siaw Chui Chai, Nor Afifi Razaob Razab, Mohd Iskandar Mohd Amin, Julianne W. Howell

**Affiliations:** 1 Occupational Therapy Programme, Centre for Rehabilitation and Special Need Studies, Faculty of Health Sciences, Universiti Kebangsaan Malaysia, Jalan Raja Muda Abdul Aziz, Kuala Lumpur, 50300 Malaysia; 2 Hand and Upper Limb Centre, Pantai Hospital Kuala Lumpur, Kuala Lumpur, Malaysia; 3 Saint Joseph, MI USA

**Keywords:** Handicraft, Fracture, Occupational therapy, Hand rehabilitation, Occupation-based intervention, Purposeful activity

## Abstract

**Background:**

Occupational Therapists use craft-making activities as therapeutic interventions to improve physical and psychological functioning of injured people. Despite the therapeutic effects, craft-making is not routinely used in hand rehabilitation as an intervention for patients with upper limb fractures. These patients often experience physical and psychosocial issues; however, without supportive evidence, therapists hesitate to integrate craft-making into upper limb rehabilitation.

**Purpose:**

This study aims to determine the effect of a conventional therapy combined with therapeutic craft-making on disability, post-traumatic stress, and physical performance in patients with lower-third forearm fractures.

**Methods:**

Priori analysis determined that 38 patients will be needed for this superiority randomized controlled trial to be conducted in a hand and upper limb rehabilitation center. Eligible participants must comprehend English, be diagnosed with lower-third forearm fracture(s) stabilized by open reduction internal fixation, and referred to therapy within 2–4 weeks of surgery. Following the CONSORT guidelines, participants will be randomly assigned to a Control (conventional therapy) group or an Intervention (conventional therapy and craft) group. Twice weekly for 6 weeks, Therapist A will provide both groups with 1-h of conventional therapy while the Intervention group will also receive 15 min of craft-making supervised by the Researcher. The primary outcome of disability will be measured with the Quick-Disabilities of Arm, Shoulder and Hand. The secondary outcome measurements include the Patient-Rated-Wrist-Evaluation; Impact of Event Scale-revised and physical performance, i.e., the Purdue Pegboard Test, AROM, and grip strength. All outcome measures will be obtained by Therapist B prior to the 1st therapy visit and after the 12th visit. Descriptive analysis will be done for the categorical and continuous data and a mixed model ANOVA for analysis of the initial and final assessment scores within and between groups.

**Results:**

This study is ongoing.

**Discussion:**

The intent of this study is to determine if therapeutic crafts have value as an intervention when used in combination with conventional therapy for patients with lower-third forearm fractures. If the value of crafts is supported, this evidence may reduce hesitancy of therapists to implement craft-making with patients referred to hand therapy after upper limb fracture.

**Conclusion:**

This study is ongoing.

**Trial registration:**

ANZCTR, ACTRN12622000150741. Retrospectively registered on 28 January 2022 https://anzctr.org.au/Trial/Registration/TrialReview.aspx?id=382676&isReview=true..

**Supplementary Information:**

The online version contains supplementary material available at 10.1186/s13063-024-08008-w.

## Introduction

### Background and rationale {6a}

Injury to the upper limb can have serious consequences on a person’s physical, psychological, social, and financial aspects of life [1–3]. Traumatic injuries of the upper limb commonly involve fractures around the wrist joint, including the lowerthird of the forearm [4–6]. These fractures are frequently stabilized by open reduction and internal fixation (ORIF) as this technique allows for early controlled mobilization and functional use of the upper limb [4, 6].

Occupational Therapists who practice hand therapy are inclined to follow a biomedical approach, which places less emphasis on the client-centered or occupation-based approach [3, 7–9]. Therapists working in medically oriented facilities as opposed to welfare or private facilities tend to implement more physical modalities, splints, soft tissue manipulation, and mobilization [2, 3, 7]. This biomedical approach requires less active patient participation, which has been observed to not directly support the patient’s psychological well-being or occupational performance [7, 10].

Since the emergence of Occupational Therapy in 1917, therapists have been using occupation (self-care, work, leisure, and daily activities) and purposeful activity as methods to rehabilitate patients [3, 10, 11]. Craft-making, classified as a purposeful activity, is commonly implemented therapeutically [11]. As the biomedical model practice developed, therapists began to incorporate physical modalities and procedures into traditional occupation-based interventions [12, 13]. In 2019, the American Occupational Therapy Association stressed the importance of purposeful activities and provided guidelines for defining and applying purposeful activities [14]. Furthermore, therapists have been encouraged to incorporate art and craft into their patient management [12].

Therapeutically, the tangible properties of craft-making are known to provide a feeling of satisfaction, hope, and pride to the maker for a long period of time [11, 15, 16], and there is evidence to support the value of craft-making implementation during short hospital stays [17]. Craft-making is well-established as a method for providing patients with the tactile and proprioceptive sensorimotor input needed to improve fine motor control [15, 18]. Besides providing motivation, craft-making is also used for its psychophysiological and emotional effects, such as reducing pain, anxiety, and depression [12, 15].

One reason that crafts may not be routinely implemented by therapist in the hospital or hand rehabilitation settings may be simply the lack of supportive evidence [11, 15, 17].

### Objectives {7}

The objective of this study is to determine the effects of therapeutic craft-making on disability, post-traumatic stress, and physical performance compared with a 6-week conventional hand therapy protocolled approach implemented for patients with lower-third forearm fractures. Our hypotheses are that patients with lower-third forearm fractures who received 6 weeks of combined conventional therapy and therapeutic craft-making in comparison to conventional therapy only will have (1) less disability as measured by the Quick-Disabilities of the Arm, Shoulder and Hand (Quick-DASH) and the Patient Rated Wrist Evaluation (PRWE); (2) less post-traumatic stress as measured by the Impact of Event Scale-Revised (IES-R); (3) better physical performance, i.e., dexterity, as measured by the Purdue Pegboard Test (PPT), and active range of motion (AROM) of the forearm, wrist, and digits as measured by a goniometer and grip strength as measured by a Jamar™ dynamometer.

### Trial design {8}

**Fig. 1:**
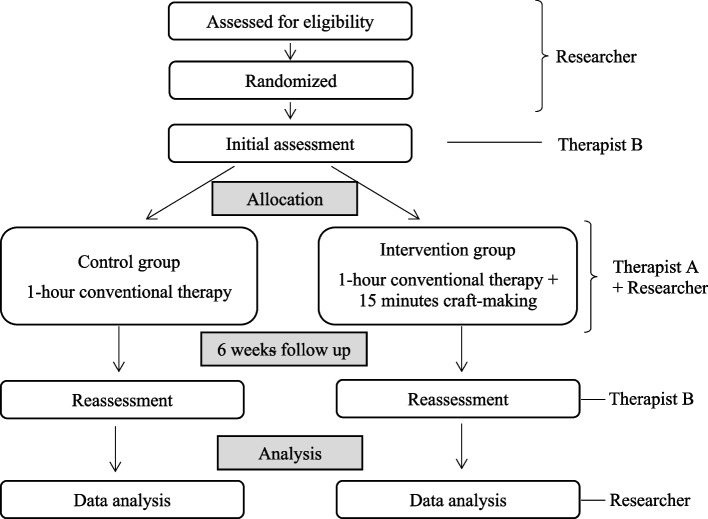
CONSORT flow diagram

This study is a superiority randomized controlled trial (RCT). SPIRIT checklist (Supplementary Material 1) was used as a guidance in developing this study and to ensure the quality of this study. The trial will be conducted and reported in accordance with CONSORT guidelines [19]. The CONSORT flow diagram for this study is shown in Fig. 1. Participants will be randomly assigned to either the Control group or the Intervention group.

## Methods

### Study setting {9}

This study will be conducted at the Hand and Upper Limb Centre, Pantai Hospital Kuala Lumpur, Malaysia.

### Eligibility criteria {10}

The inclusion criteria are patients with (1) lower-third of the forearm extra-articular, partial articular, and/or complete articular individual or combined fractures of the radius, ulna or distal radioulnar, and/or radiocarpal joint, (2) fracture(s) stabilized with ORIF technique within 2–4 weeks of referral, and (3) the ability to read and communicate in English. The exclusion criteria include (1) diagnoses of rheumatoid arthritis, multiple limb or body fractures, carpal fracture/injury, or associated peripheral nerve or tendon injury, and (2) patients with a history of cognitive or psychological impairment. For this RCT, there is no age limit to participate; however, if there is a large variance in age, sub-analysis will be performed.

### Who will obtain informed consent? {26a}

During the first therapy visit, the Researcher will brief participants regarding the study and obtain informed consent. Participants will need to read the information sheet and sign the informed consent form before participating in this study.

### Additional consent provisions for collection and use of participant data and biological specimens {26b}

Not applicable as no biological specimens will be collected as part of this study.

## Interventions

### Explanation for the choice of comparators {6b}

#### Control group

Participants in the Control group will receive 1-h of conventional therapy described in the protocol (Table 1) including hot/cold packs, ultrasound, joint mobilization, AROM exercises including tendon gliding, passive stretching, scar massage, and therapeutic exercise to improve pinch, grasp, and forearm strength. All conventional therapy sessions will be conducted by Therapist A (Fig. 1).

**Table 1:** Conventional therapy protocol for both the Control and Intervention groups

Week	Therapy
1	Heat/cryotherapy Active mobilization of wrist and forearm Mobilization of uninvolved joints Light grasp and pinch Tendon gliding Ultrasound Scar massage
2	Heat/cryotherapy Gentle stretching of wrist and forearm Mobilization of uninvolved joints Grasp and minimally resistive activities Ultrasound Scar massage
3	Heat/cryotherapy Passive stretching to gain full range of motion of wrist and forearm Light forearm strengthening Moderate resistive grasp and pinch Ultrasound Scar massage
4	Heat therapy Passive stretching to gain full range of motion of wrist and forearm Forearm strengthening Moderate resistive grasp and pinch Ultrasound Scar massage
5	Heat therapy Passive stretching to gain full range of motion of wrist and forearm Forearm strengthening Functional activities—pinch tree and EZ board Ultrasound Scar massage
6	Heat therapy Passive stretching to gain full range of motion of wrist and forearm Forearm strengthening Functional activities—pinch tree and EZ board Ultrasound Scar massage

### Intervention description {11a}

#### Intervention group

**Fig. 2:**
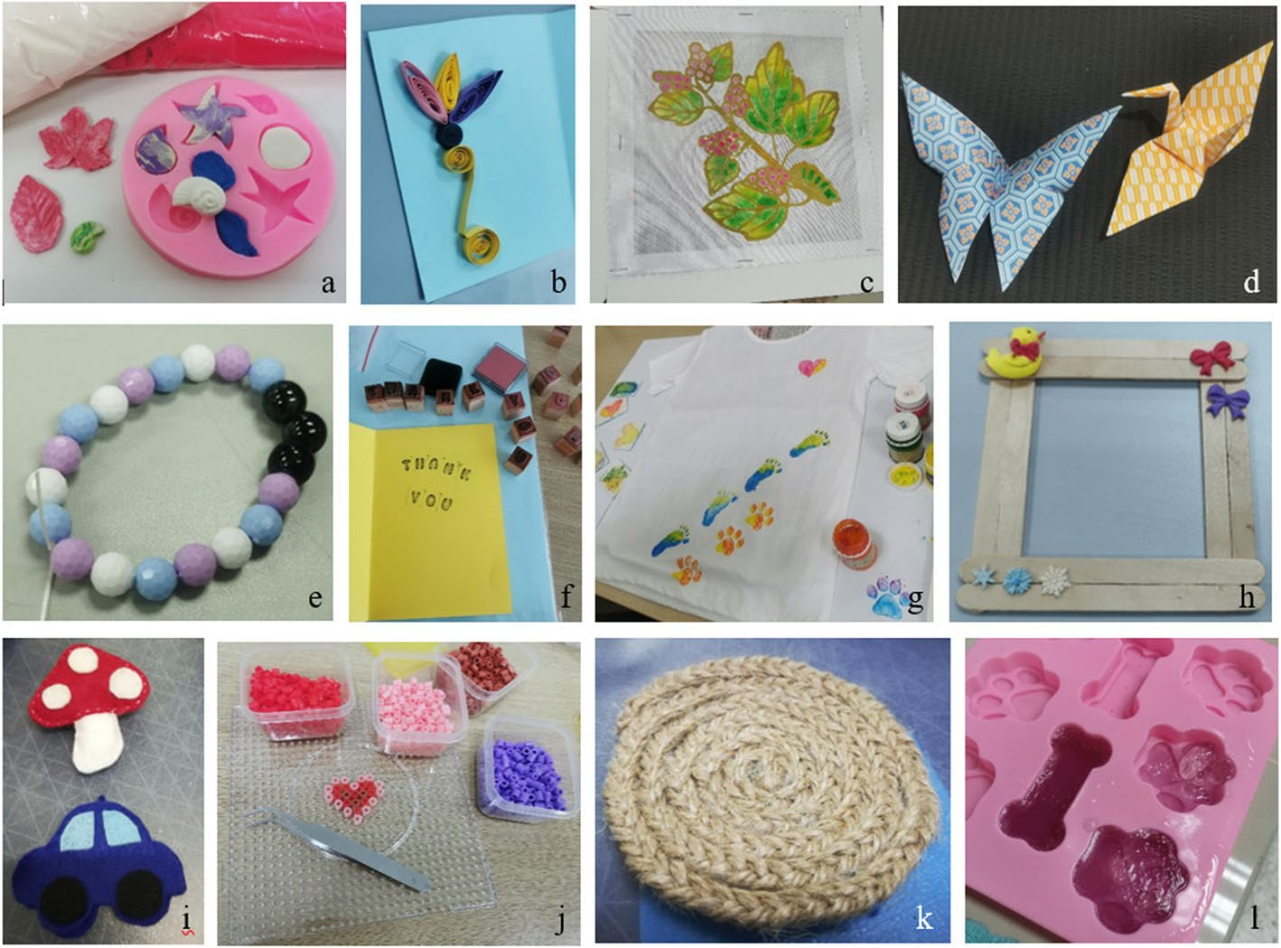
Examples of crafts to be performed by participants in the Intervention group: **a** air dry clay art, **b** quilling, **c** batik painting, **d** origami, **e** threading beads, **f** card making, **g** cloth stamping, **h** wooden frame design, **i** sewing, **j** Perler beading, **k** braiding and cup mat, and **l** soap making

During 1-h of conventional therapy, Therapist A will follow the protocol described in Table 1. After conventional therapy, the Researcher will provide an additional 15 min of therapeutic craft-making activities as delineated in the template for intervention description and replication (TIDieR) in Table 2. These activities will be done in a separate room and the participants will be given a 5–10-min break between conventional therapy and the therapeutic craft-making sessions. The 6 weeks of therapeutic craft-making is outlined in protocol format in Table 3. This craft-making protocol was developed through activity analysis completed by six Occupational Therapy students explicitly for this study and will be conducted according to the list of craft-making protocol developed earlier (Table 3 and Fig. 2).

**Table 2:** Template for therapeutic craft-making intervention description and replication (TIDieR) checklist (Supplementary Material 2)

	Therapeutic craft-making
Why	A method to provide sensorimotor input for improvement of fine motor skills and regulate psychophysiological and emotional response
What	All materials are available in craft shops or online marketplaces. These included air dry clay, silicone mold, papers, quilling papers, paper glue, quilling pen, batik cloth, cloth dyes, dropper, origami papers, beads, elastic thread, stamps, stamp pads, ice-cream sticks, hot glue, Perler beads, Perler board, forceps, strings, felt cloths, cottons, needles, metal ruler, soap base, perfume, and soap dye
Who provided	The Researcher
How	1 on 1 individually
Where	A designated room away from the general treatment area
When and how much	After 1-h of conventional therapy, twice weekly for 6 weeks for approximately 15 min per session
Tailoring	Craft will be adapted to individual’s hand function and performance Simplification of tasks or assistance will be provided if participant is struggling to complete the craft

**Table 3:** Craft-making protocol for intervention group

Week	Craft	Steps
1	Air dry clay art	Open package of clay and take out a small amount of clay Knead clay with fingers Insert clay into mold Even out the clay using fingertips Remove excess clay
	Quilling	Pour a small amount of glue on unwanted paper Choose a piece of quilling paper and insert one end into the quilling pen Turn the pen to roll the paper until the end Shape quilled paper according to design Place the quilled paper on glue to apply glue Glue quilled paper on card, press until it firmly sticks
2	Batik painting	Dominant hand Dip paintbrush in fabric dye and transfer to palette Dilute dye with water Color the batik Clean paint brush by dipping it into water Non-dominant hand Open dropper’s cap Point the tip of the dropper onto the desire area on the batik Squeeze dropper Use the dropper point to spread the color around
	Origami	Take a piece of origami paper Flip and fold paper according to the steps Use fingertip to press on folded sides
3	Threading beads	Cut elastic thread and tie a knot on one end of the thread Take beads from packet/container Treading beads into the other end of the thread Tie both ends together once finished Cut excess thread
	Card making	Cut A4 paper into half and trim edges Fold the paper in half and cut out the design Pick up alphabet stamps Stamp on ink pad then stamp on the card to finish sentences Glue the A4 paper to card paper with quilling
4	Cloth stamping	Fold newspaper in half and insert it into inner layer of the shirt Pick up a stamp and apply fabric dye using paintbrush Put paintbrush into a cup of water Turn over the stamp and put on the cloth Press on stamp for 5 s Carefully remove stamp
	Wooden frame design	Apply hot glue to the end of stick Glue wooden sticks in parallel to make a square frame Apply instant glue on decorative clay Glue decorative clay on to the frame
5	Sewing	Insert thread into the hole of a needle Tie a knot at the end of the thread Match the layer of design and sew the edge of the design until ¾ complete Put cotton into the opening Sew the product until finish Tie a few knots and trim the excess thread
	Perler beading	Pick a design and take a Perler board Use a finger tweezer to pick up Perler beads Place beads on board according to design Carefully place the iron paper against the completed beaddesign Iron Perler beads until it sticks together Flip the beads to the other side and continue to iron Remove Perler from iron paper
6	Braiding and cup mat	Find the midpoint of a rope and tie a knot at the middle of another rope Start braiding from top Tape to secure the top of the rope on table Continue braiding until finish Use hot glue to secure the finished end Curl the rope, glue, and then roll the braided rope into a circular shape
	Soap	Cut the soap base into small pieces using a metal ruler Put soap pieces into a metal cup Heat the metal cup in hot water Put a drop of soap dye and fragrance into melting soap Stir soap slowly using a stick Pour mixture into the mold Spray alcohol on the soap to reduce bubbles

The copyright of this craft-making protocol for lower-third forearm fracture is owned by Universiti Kebangsaan Malaysia

### Criteria for discontinuing or modifying allocated interventions {11b}

Given some of the participants may show different rates of improvement and recovery after surgery, each craft will be graded and modified according to the participant’s hand dominance and his/her ability to use the injured hand. The Researcher will take precautions in selecting safe materials and activities, yet some participants may need assistance with sharp tools such as scissors.

### Strategies to improve adherence to interventions {11c}

The Researcher will track participant attendance to ensure all therapy sessions are completed before Therapist B does the final assessment. If a participant fails to attend a session, the Researcher will phone the participant to reschedule within the timeframe of the study.

### Relevant concomitant care permitted or prohibited during the trial {11d}

Participants will be allowed to consume any prescribed medication, including analgesics, during the study. If, however, analgesic consumption exceeds more than that prescribed while participating, this will be documented by Therapist A and the Researcher. Participants will be excluded if other Occupational therapy or Physiotherapy for the injured hand is initiated while participating in this 6-week study. During the study, Intervention group participants will be allowed to replicate the protocol craft-making activities at home.

#### Provision for post-trial care {30}

Participants in both the Control and Intervention groups will be allowed to continue therapy as necessary after completing the 6-week study.

#### Outcomes {12}

The primary outcome implemented in this study is the Quick-DASH (Disabilities of the Arm, Shoulder and Hand) to assess upper limb disability. The secondary outcomes include the Patient Rated Wrist Evaluation (PRWE), which is specific for evaluating wrist disability and the Impact of Event Scale-Revised (IES-R) for rating post-traumatic stress. Additional secondary outcomes include components of physical performance (measured by)—dexterity (Purdue Pegboard Test), AROM of forearm, wrist and fingers (goniometer), and grip strength (Jamar™ dynamometer).

### Participant timeline {13}

Participants will be required to attend therapy twice weekly for 6 weeks for a total of 12 therapy sessions. The enrollment, intervention, and assessment began in July 2021 and was expected to end in December 2023 (Table 4).

**Table 4:**
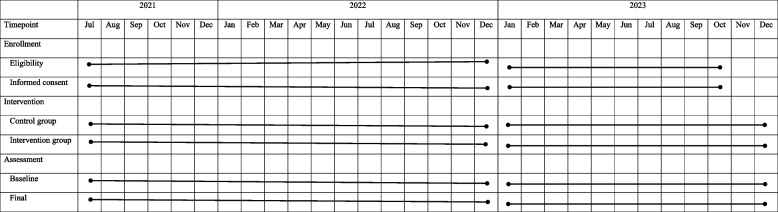
Schedule of enrollment, interventions, and assessments

#### Sample size {14}

In regard to the primary outcome of this study, the sample size required was calculated with a G* power sample size calculator. Analysis was done using ANOVA repeated measures, within-between interaction in *F* tests family. A priori type of power analysis was used to compute the required sample size. Analysis was done with Cohen-f effect size at 0.25 for a medium effect, alpha error at 0.05, and 80% power. There are two groups (Control and Intervention) with two sets of measurements (initial baseline and final) to be compared. Correlation among repeated measure was set at 0.5 and the non-sphericity correction was 1.0. The result of priori analysis indicates a total of 34 participants will be needed; however, a 10% dropout rate (4 participants) is anticipated bringing the total sample size required to 38 participants.

### Recruitment {15}

All patients with lower-third forearm fractures stabilized by ORIF, who are referred to Occupational Therapy between 2–4 weeks after ORIF and meet the inclusion criteria will be recruited.

## Assignment of intervention: allocation

### Sequence generation {16a}

Group allocation will be done using a random draw method requiring the participant to draw from a large envelope holding two smaller opaque-sealed envelopes each containing a different group assignment. This study will not use an allocation ratio.

### Concealment mechanism {16b}

There will be a large envelope with two opaque-sealed envelopes containing a slip of paper labeled as either Control or Intervention group. The larger envelope will be presented to each participant with instruction to randomly pick a sealed envelope.

### Implementation {16c}

Randomization is done by the Researcher on the initial therapy visit.

## Assignment of intervention: blinding

### Who will be blinded {17a}

Therapist A and Therapist B will be blinded to the participant’s group allocation. The Researcher will not be blinded as the Researcher will be involved in the craft-making sessions. Similarly, the participants once assigned to a group will no longer be blinded to the other group’s intervention. To ensure that Therapist A and Therapist B remain blinded over the 6-week study, the Researcher will continually remind each participant not to reveal his/her group allocation to Therapist A or B.

### Procedure for unblinding if needed {17b}

There is no known circumstance or procedure that would require revealing a participant’s allocation.

## Data collection and management

### Plans for assessment and collection of outcomes {18a}

Prior to the first therapy session, Therapist B will do the initial baseline assessment. During the assessment, each participant will be interviewed by Therapist B to obtain his/her demographic data and pertinent medical history. After obtaining this information, the outcome assessments related to disability, physical performance, and post-traumatic stress will be done. Therapist B will also do the 6-week reassessment after completion of the 12th therapy session.

### Outcome measures

#### Quick-Disabilities of the Arm, Shoulder and Hand (DASH)

The Quick-DASH is a shortened version of the original DASH, and both versions are self-report questionnaires [20]. The Quick-DASH has 11 items that are useful for assessing physical function, symptoms, and quality of life in people with upper limb musculoskeletal disorders. The calculated score for the Quick-DASH, using the formula below, ranges between 0 and 100 with a higher score an indication of greater disability [21, 22]. The minimal clinically important difference for Quick-DASH is 12.85 at 90% confidence level [23].

Quick DASH score formula:

$$\mathrm{Total\ score}=\left(\left[\frac{\mathrm{sum\ of\ }n\mathrm{\ responses}}{n}\right]-1\right)\times 25$$

#### Patient-Rated Wrist Evaluation (PRWE)

The PRWE is a self-report questionnaire designed to measure wrist pain and disability experienced during activities of daily living after wrist injury. The PRWE has 15 items that are divided into two subscales: pain (5 items) and function (10 items) [20]. The total score is calculated using a provided formula with a higher score an indication of greater disability [22, 24]. The minimal clinically important difference for PRWE for patient with distal radius fractures is 11.5 points for a 95% confidence level [25].

#### Impact of Event Scale-Revised (IES-R)

The IES-R is also a self-report questionnaire used to assess a person’s response to traumatic events. The IES-R has 22 items divided into three subscales: intrusion, avoidance, and hyperarousal. The IES-R measures the frequency of symptoms occurrence over the past week. The results of the subscales are added for a total score that can range between 0 and 88 with a higher score an indication of more post-traumatic stress [26, 27].

#### Purdue Pegboard Test (PPT)

The PPT is a performance-based assessment for measuring fine and gross motor dexterity of the hand and fingers. There are five subtests: (1) Right hand, (2) Left hand, (3) Both hand, (4) Total of Right hand + Left hand + Both hand (not an actual test but a summation of scores of the first three subtests), and (5) Assembly. Each subtest will be performed by the participant three times and the average score calculated. The validity coefficients of the PPT ranges between 0.70 and 0.76 depending on the demographic characteristics of the participant [28].

#### Goniometry

A standard 6-in plastic goniometer will be used to measure active forearm supination and pronation as well as wrist flexion, extension, and radial and ulnar deviation. To measure AROM of the finger and thumb MCP and IP joints, a flat metal finger goniometer will be used. Active forearm rotation will be measured by stabilizing the participant’s elbow at his/her side with the elbow flexed to a 90° angle. Active radial and ulnar deviation motion will be measured by placing the participant’s hand palm down on the tabletop or if forearm pronation is limited the participant will rest his/her elbow on the tabletop. Active wrist flexion and extension as well as digital motion will be measured with the participant’s elbow on the tabletop in a hand up position. AROM measurements of the digits will be recorded as total active motion (TAM) calculated in a standard manner described by American Society for Surgery of the Hand using formula below [29].

$$\mathrm{Finger\ TAM }= (\mathrm{MP\ flexion }+\mathrm{ PIP\ flexion }+\mathrm{ DIP\ flexion}) - (\mathrm{MP\ extension\ deficit }+\mathrm{ PIP\ extension\ deficit }+\mathrm{ DIP\ extension\ deficit})$$

$$\mathrm{Thumb\ TAM }= (\mathrm{MP\ flexion }+\mathrm{ IP\ flexion}) - (\mathrm{MP\ extension\ deficit }+\mathrm{ IP\ extension\ deficit})$$

#### Dynamometry

Grip strength will be measured using a Jamar™ dynamometer with the handle position set on the second notch while the participant is seated with his/her elbow flexed to 90° and stabilized beside the side of the body as recommended by the American Society of Hand Therapists [30]. Therapist B will ask the participant to squeeze the handle as hard as he/she can for 3 s while the reading is being obtained. The grip strength test will be performed three times beginning with the non-affected hand then alternating to the affected hand. Participants will be advised not to exceed their comfort level and not to cause pain when squeezing with their affected hand. For participants who are not able to generate any force with their affected hand, 0 kg will be recorded. The grip strength score is the average of three trials for each hand recorded in kilograms.

### Plans to promote participant retention and complete follow-up {18b}

There are no plans to promote participant retention or to complete follow-up. Follow-up data will not be collected for participants who discontinue or deviate from intervention protocols.

### Data management {19}

Once the data is gathered and the study is completed, the raw materials will be kept in a locked file cabinet in the office of the Researcher. Electronic data will be kept secure and password protected. Only the Researcher will have access to the data. All materials will be shredded and discarded 5 years after completion of this study.

### Confidentiality {27}

Data obtained in this study will be kept and handled confidentially, in accordance with applicable laws and/or regulations. The participant will remain anonymous in any report of this data.

### Plans for collection, laboratory evaluation and storage of biological specimens for genetic or molecular analysis in this trial/future use {33}

Not applicable as no biological specimens will be collected for this study.

## Statistical methods

### Statistical methods for primary and secondary outcomes {20a}

All data will be analyzed using the IBM Statistical Product and Service Solutions (IBM SPSS). Descriptive analysis for categorical data such as gender, marital status, hand dominance, type of wrist fracture, and educational level will be expressed in frequency, percentage, and proportion. Descriptive statistics will be used to analyze continuous data including age, Quick-DASH score, PRWE score, IES-R score, PPT score, AROM of the forearm and wrist, TAM for the digits, and grip strength. These results will be expressed as mean, standard deviation, minimum and maximum if the data is normally distributed or as median and interquartile range. If the data is not normally distributed, sub-analysis will be conducted to determine the outlier and the outlier will be extracted from the data set if it shows significant influence. A mixed model ANOVA will be used to test the primary outcome of this study by evaluating the mean difference between and within groups of the Quick-DASH scores obtained at baseline and after the 12th therapy session. The difference in the initial and final PRWE score, IES-R score, PPT score, AROM of the forearm and wrist, and TAM of the digits and grip strength will be analyzed using a mixed model ANOVA. Depending on the results of the aforementioned analyses, other sub-analysis will be performed as necessary.

### Interim analyses {21b}

There are no interim analyses or stopping guidelines for this study.

### Methods for additional analyses (e.g., subgroup analyses) {20b}

There are no additional analyses planned for this study.

### Methods in analysis to handle protocol non-adherence and any statistical methods to handle missing data {20c}

Missing data will be replaced with the mean imputation method. Intention-to-treat analysis will be used for participants that are non-adherent.

### Plans to give access to the full protocol, participant level-data and statistical code {31c}

There is no plan for granting public access to the participant-level dataset or statistical code.

## Oversight and monitoring

### Composition of the coordinating center and trial steering committee {5d}

This study does not have a coordinating center or trial steering committee.

### Composition of the data monitoring committee, its role, and reporting structure {21a}

This study does not have a monitoring committee.

### Adverse event reporting and harms {22}

The protocol and interventions used in this study are very low to no risk in terms of adverse events or harm. Should any adverse event or harm occur, these will be reported to the Researcher’s supervisors and ethical committee.

### Frequency and plans for auditing trial conduct {23}

There are no plans to perform an auditing trial of conduct.

### Plans for communicating important protocol amendments to relevant parties (e.g., trial participants, ethical committees) {25}

There are no important protocol amendments for this study.

#### Dissemination plans {31a}

The results of this study will be disseminated through publication in journals.

## Discussion

Since the introduction of the biomedical model to Occupational Therapy, crafts have rarely been used as a therapeutic intervention upper limb rehabilitation. Because of this, therapists not only lack awareness but also functional knowledge about how to integrate craft-making into practice to support the physical, psychological, and social needs of patients with upper limb injuries [9, 31]. The results of this study may bring attention to the therapeutic effect of craft-making while rehabilitating patients after lower-third forearm fractures. Since a majority of hand therapists are Occupational Therapists [7, 9], integration of therapeutic craft-making activities into the rehabilitation of patients with upper limb disorders would align with the nature of Occupational Therapy profession, which embraces the use of occupation-based and purposeful activities as treatment interventions [9, 11].

## Study limitations

- The craft-making protocol implemented in this study was designed by activity analysis for patients with the diagnosis of lower-third forearm fractures. It is unknown if the findings of this study can be generalized to other forearm, wrist, or upper limb injuries.
- The use of materials with different sizes and textures can potentially change the complexity of the task and consequently affect the grading of these activities. Therefore, it is unknown if the findings of this study can be generalized if different crafts or materials are used.
- This small sample of participants recruited from a single centre may not represent all patients that experience lower-third forearm fractures.
- Self-report outcome questionnaires depend on participant perception as do the outcomes achieved in usual practice. Some perceptions are beyond the control of this study just as experienced in usual practice.
- Knowing that surgical methods and technical expertise in fracture fixation vary as well has a person’s rate of healing, the protocols used in this study were established to provide some control over these variables.

## Trial status

There is only one protocol version for this study that began on July 12, 2021. This study is currently in the data collection phase where participant recruitment and implementation of the intervention is ongoing.

### Supplementary Information

**Supplementary Material 1.**

** Supplementary Material 2.**

## Data Availability

The data set supporting the conclusion of this article is currently unavailable as participant recruitment and intervention is ongoing.

## Abbreviations

ORIF: Open reduction and internal fixation
OT: Occupational Therapists
Quick-DASH: Quick-Disabilities of the Arm, Shoulder and Hand
PRWE: Patient-Rated Wrist Evaluation
IES-R: Impact of Event Scale-Revised
PPT: Purdue Pegboard Test
AROM: Active range of motion
TAM: Total active motion
